# Mesenchymal stem cell-derived exosomes as new tools for delivery of miRNAs in the treatment of cancer

**DOI:** 10.3389/fbioe.2022.956563

**Published:** 2022-09-26

**Authors:** Aysegul Dalmizrak, Ozlem Dalmizrak

**Affiliations:** ^1^ Department of Medical Biology, Faculty of Medicine, Balıkesir University, Balıkesir, Turkey; ^2^ Department of Medical Biochemistry, Faculty of Medicine, Near East University, Nicosia, Mersin, Turkey

**Keywords:** mesenchymal stem cells, exosomes, micro RNA, cancer therapy, cell free therapy

## Abstract

Although ongoing medical research is working to find a cure for a variety of cancers, it continues to be one of the major causes of death worldwide. Chemotherapy and immunotherapy, as well as surgical intervention and radiation therapy, are critical components of cancer treatment. Most anti-cancer drugs are given systemically and distribute not just to tumor tissues but also to normal tissues, where they may cause side effects. Furthermore, because anti-cancer drugs have a low delivery efficiency, some tumors do not respond to them. As a result, tumor-targeted drug delivery is critical for improving the safety and efficacy of anti-cancer treatment. Exosomes are microscopic extracellular vesicles that cells produce to communicate with one another. MicroRNA (miRNA), long non-coding RNA (lncRNA), small interfering RNA (siRNA), DNA, protein, and lipids are among the therapeutic cargos found in exosomes. Recently, several studies have focused on miRNAs as a potential therapeutic element for the treatment of cancer. Mesenchymal stem cells (MSC) have been known to have angiogenic, anti-apoptotic, anti-inflammatory and immunomodulatory effects. Exosomes derived from MSCs are gaining popularity as a non-cellular alternative to MSC-based therapy, as this method avoids unwanted lineage differentiation. Therefore more research have focused on transferring miRNAs to mesenchymal stem cells (MSC) and targeting miRNA-loaded exosomes to cancer cells. Here, we initially gave an overview of the characteristics and potentials of MSC as well as the use of MSC-derived exosomes in cancer therapy. Finally, we emphasized the utilization of MSC-derived exosomes for miRNA delivery in the treatment of cancer.

## Introduction

The human body comprises a variety of cell types that make up tissues and organs with distinct functions that contribute to long-term survival. Long ago, it was discovered that differentiated cells in several tissues, such as the skin, intestinal epithelium, and blood, have a short lifecycle and are unable to self-renew (Watt and Driskell, 2010). Stem cells may self-renew and have the ability to differentiate into a variety of cell types in an organism. This discovery gave rise to the concept of stem cells, which are small unspecialized cells in the human body that lack a variety of phenotypic features observed in adult tissues and are used to maintain static and temporary cell types (Alvarez et al., 2012). Embryonic and non-embryonic stem cells (somatic stem cells) are the two basic types of stem cells. Embryonic stem cells are pluripotent, but somatic stem cells, mesenchymal stem cells (MSCs), for example, are multipotent stem cells (Singh et al., 2016). Because of their unique properties, such as self-renewal and the ability to differentiate into a variety of cell types, MSCs are among the most studied stem cells (Pittenger et al., 2019).

Friedenstein and colleagues were the first to isolate and define MSCs from bone marrow as adherent, highly replicative cells that can differentiate into mesodermal lineages such as osteoblasts, chondrocytes, adipocytes, and hematopoietic stroma (Friedenstein et al., 1966). In addition to bone marrow, MSCs can be isolated from variety of tissues (da Silva Meirelles et al., 2006). According to the International Society for Cellular Therapy (ISCT), MSCs must fulfill three minimal conditions; (1) adherence to plastic surface when cultured *in vitro*, (2) expression of the surface antigens CD73, CD90, and CD105, and absence of CD34, CD45, CD14 or CD11b, CD79α or CD19, and HLA-DR, (3) ability to form several mesodermal cell types, such as adipocytes, chondrocytes, and osteoblasts when cultured *in vitro* under appropriate conditions (Dominici et al., 2006).

MSCs are attractive therapeutic targets for a variety of disorders, including cancer treatment and tissue regeneration, because of their versatility and ability to self-renew. MSCs have undeniable medical potential; yet, their capacity to develop into tumor-associated fibroblasts (Mishra et al., 2008; Miyazaki et al., 2021), which promote tumor growth via their secretome (Liang W. et al., 2021), and resistance to apoptosis, makes them potentially dangerous (Bellagamba et al., 2016). MSCs have not been successfully used in anticancer therapy because of their contradictory involvement in cancer progression and regression. To effectively harness MSCs’ therapeutic potential, it is critical to understand their underlying molecular pathways.

Exosomes are extracellular vesicles (EVs) produced by eukaryotic cells that serve as carriers for the transfer of membrane and cytosolic proteins, lipids, and RNA between cells, making them a key component of intercellular communication (Raposo and Stoorvogel, 2013). These membrane–bounded vesicles can be divided into three subtypes, exosomes (50–150 nm), microvesicles (100–1,1000 nm), and apoptotic bodies (500–5,5000 nm) (Doyle and Wang, 2019). Exosomes and other EVs have been found in a variety of tissues and biological fluids, including urine, blood, and cerebrospinal fluid. MicroRNAs (miRNAs) and proteins are mostly found in exosomes, which are enclosed by a lipid bilayer membrane (Zhang et al., 2018). Exosomes also contain other RNA types such as nucleolar RNA, long noncoding RNA, and ribosomal RNA, as well as DNA fragments (Sato-Kuwabara et al., 2015). Studies have shown that released exosomes can be guided to other cells *via* proteins found on cell surfaces (Neviani and Fabbri, 2015).

Exosomes derived from MSCs have been shown to possess potential benefits for the management of several pathological conditions, including cancer. MSC-derived exosomes have almost all of the properties of the original cells, in terms of paracrine effects and immunomodulatory functions. Recently, loading MSC-derived exosomes with defined cargos such as miRNAs has been suggested to be a promising strategy for the treatment of different diseases. Even more, genetically engineered miRNAs can be used in correcting the pathways disrupted in cancer. In the present review we discuss the function of exosomal miRNAs derived from MSCs in different type of cancers.

## Biological functions of mesenchymal stem cells

MSCs share many properties with other stem cells, including robust self-renewal and multidirectional differentiation capacity. In previous studies, MSCs have been shown to be capable of differentiating into cells of the mesodermal, ectodermal, and endodermal lineages (Dominici et al., 2006; Paunescu et al., 2007). MSCs can regulate the immune system by interacting with immune cells and also have paracrine effects. Furthermore, because MSCs have a low immunogenicity, allograft matching requirements are less stringent, and immunological rejection is less likely. MSCs can thus be used as ideal seed cells for repairing tissue and organ damage caused by aging and pathological changes, and they also have broad clinical applications in the treatment of autoimmune diseases, inflammation-related diseases, and cancer (Farini et al., 2014).

MSCs exert their immunomodulatory activity through interacting with immune cells in both the native and acquired immune systems. First, MSCs decrease natural killer (NK) cell proliferation, cytotoxicity, and cytokine secretion by secreting prostaglandin E2 (PGE2), indoleamine 2,3-dioxygenase (IDO), and soluble human leukocyte antigen G5 (sHLA-G5) (Galland et al., 2017). MSCs can also influence the development of dendritic cells (DCs) by suppressing monocyte differentiation into DCs (Jung et al., 2007). MSCs can limit the expression of tumour necrosis factor (TNF) (Yan et al., 2018) and enhance the expression of interleukin 10 (IL-10) (Selleri et al., 2013) by DCs, which is likewise regulated by PGE2. MSCs also decrease the ability of naïve T cells to induce Th1 differentiation (Consentius et al., 2015), ultimately leading to immunosuppression.

In 2008, Le Blanc and Davies reported success in the treatment of graft versus host disease (GVHD) with allogeneic, semicompatible, and mismatched bone marrow-derived MSC transplantation, indicating that a strict match was not necessary in the treatment of GVHD with MSCs (Le Blanc and Davies, 2015). The low immunogenicity of MSCs is crucial to the success of allogeneic MSC transplantation in preclinical and clinical settings. MSCs express major histocompatibility complex class I (MHC I) and lymphocyte function-associated antigen (LFA-3) on a constitutive manner, but only following stimulation with interferon-gamma they express MHC II and intercellular adhesion molecule (ICAM) (Tse, et al., 2003). Furthermore, MSCs do not stimulate the proliferation of peripheral blood mononuclear cells (PBMCs) showing low immunogenicity characteristics (Parys et al., 2017). Additionally, MSCs have the ability to significantly reduce the proliferation of activated T cells and interferon-gamma has a vital role in this process (Chinnadurai et al., 2014).

MSCs can migrate to the site of a lesion in a variety of illnesses, including inflammation, tissue damage, and tumors (Nitzsche et al., 2017). Several cell adhesion molecules and chemokine receptors expressed by MSCs influence their migration to the lesion site, and MSC-targeted migration to the lesion site is referred to as “homing” of MSCs (Naji et al., 2019) which is a multistep process that includes activation, adhesion, and migration (De Becker and Riet, 2016). First, inflammatory cytokines generated by inflamed or wounded tissues activate vascular cell adhesion molecule-1 (VCAM-1) on the surface of endothelial cells and α4β1 integrin (VLA-4) on the surface of MSCs, trapping MSCs on the endothelial cell surface (Uchibori et al., 2013). Following that, many growth factors generated by inflammation or damaged tissues might bind to MSC receptors and increase MSC adherence to endothelial cells (Lejmi et al., 2015). Finally, MSCs express matrix metalloproteinase 2 (MMP-2) and membrane type-1-MMP (MT-1-MMP), which activate proteasomes that breakdown the extracellular matrix and assist MSCs migrate across the basement membrane to the lesion site (Ries et al., 2007).

## Mesenchymal stem cells in cancer therapy

**FIGURE 1:**
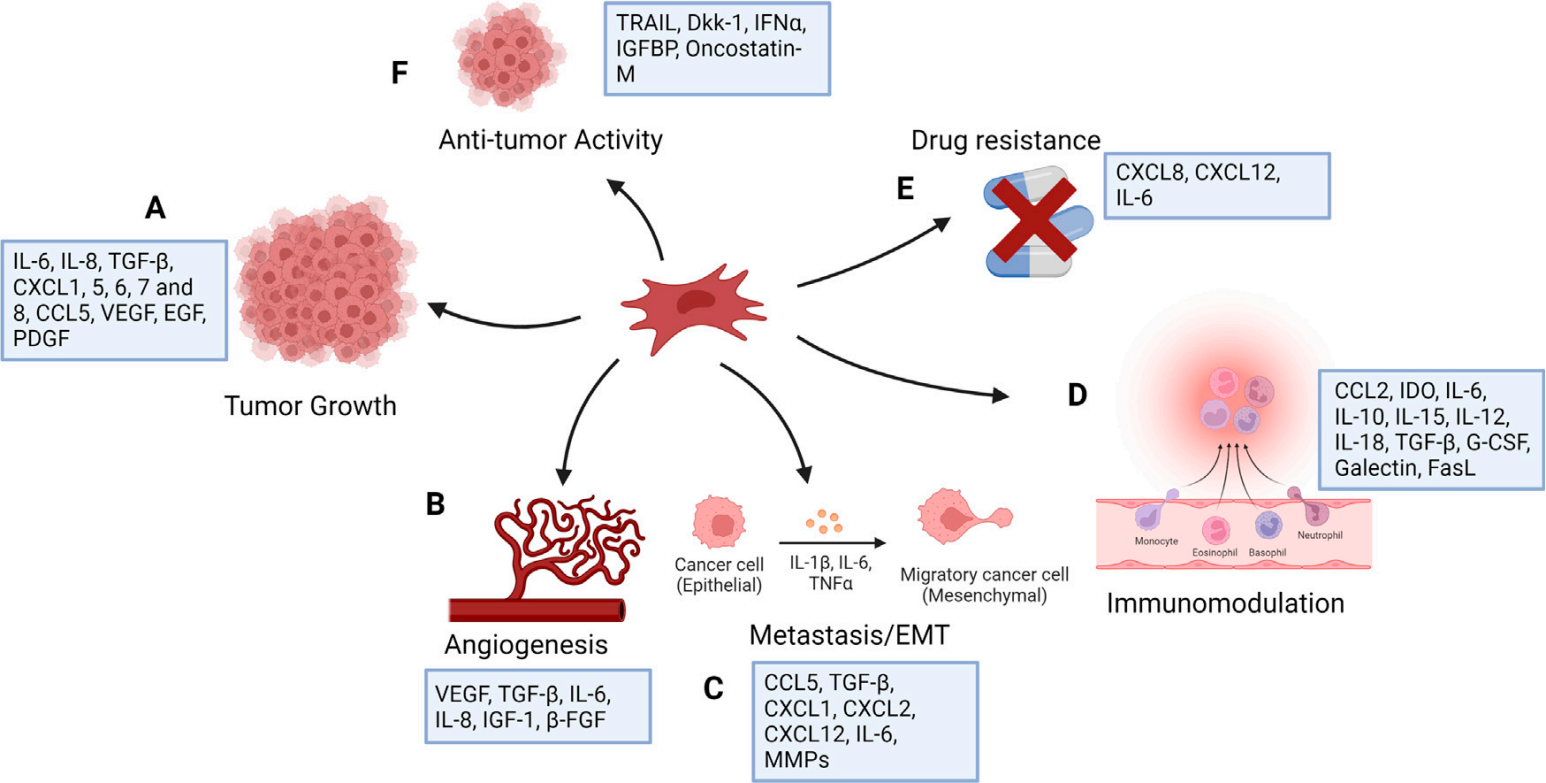
Functions of mesenchymal stem cells in cancer (created with BioRender). MSCs have number of effects on tumor cells, mostly increasing tumor growth as a result of their function in controlling inflammation and tissue repair. They affect tumor cell survival and stemness **(A)** and contribute to angiogenesis **(B)** by producing angiogenic factors. MSCs stimulate tumor cell motility, epithelial mesenchymal transition (EMT), and metastasis **(C)**, and secrete chemokines, including CXCL1, CXCL2, and CXCL12, and cytokines, including IL-6 and several matrix metalloproteinases (MMPs), which degrade the extracellular matrix to facilitate tumor cell migration. They show immunomodulatory function **(D)** and can induce drug resistance **(E)**. MSCs are generally pro-tumorigenic, however research has suggested that they may also have anti-tumor properties **(F)**.

Many mediators have been identified in the cross-talk between MSCs, the tumor microenvironment, and tumor cells. By triggering numerous signaling pathways, MSCs have different roles on the cells in the tumor microenvironment. MSCs can block Wnt signaling by regulating the Dickkopf-related protein 1 (DKK1) secreted by tumor cells, downregulating c-Myc and Cyclin D2 and upregulating the expression of P21CIP1 and P27KIP1, resulting to tumor cell suppression (Qiao et al., 2008; Zhu et al., 2009). By suppressing angiogenesis, naive MSCs can cause vascular endothelial cells to die (Otsu et al., 2009). On the contrary, MSCs have been shown to be linked to increased metastasis, tumorigenesis, and recurrence of tumors by producing cancer stem cells (CSCs) (Liu et al., 2011). MSCs also produce chemokines such as CXCR4 (Corcoran et al., 2008), CCL5 (Karnoub et al., 2007), ICAMs (Tsukamoto et al., 2012), and VCAMs (Hu et al., 2012). Breast cancer cells induce mesenchymal stem cells to secrete the chemokine CCL5, which subsequently acts in a paracrine manner on the cancer cells to promote motility, invasion, and metastasis (Karnoub et al., 2007). MSCs obtained from mouse lymphomas produce CCL2 and enhance cancer cell proliferation as well as the recruitment of immunosuppressive cells to lymphoid organs (Ren et al., 2012). MSCs originating from breast cancer tissues also produce some immunosuppressive mediators such as IL-4, TGF-β, and IL-10 (Razmkhah et al., 2011). Although the majority of studies aimed at using MSCs in cancer therapy have focused on their tumor-suppressing capabilities, these cells may potentially stimulate tumor progression by increasing metastasis, tumor angiogenesis, epithelial–mesenchymal transition, and disrupting immune surveillance (Hmadcha et al., 2020) (Figure 1). These unfavorable effects may appear depending on the number of MSCs injected, their source or origin, differentiation level, and tumor type, As a result, restrictions in MSC-based cancer therapy should be considered, and more research is needed to assess the safety and efficacy of such a therapeutic approach in the treatment of cancer.

## Exosomes as drug carriers

Exosomes are more commonly used as drug delivery vehicles because of their transport capabilities in delivering functional content to specific cells. Some natural exosomes can be used as therapeutic agents because they contain endogenous anti-tumor biomolecules. Furthermore, bioengineered exosomes with extra required payloads and targeting specificity offer more promise in cancer treatment. In contrast to other regularly used drug delivery vehicles (e.g., liposomes), bioengineered exosomes have intrinsic targeting capabilities, low immunogenicity, high modification flexibility, and biological barrier permeability (Walker et al., 2019).

Different methods are currently being employed for the purification of exosomes, such as differential ultracentrifugation, density gradient ultracentrifugation, size exclusion chromatography, etc. (Wang et al., 2021). For isolation, the International Society for Extracellular Vesicles (ISEV) has established detailed guidelines. However, none of the methods were able to accomplish absolute purification, or total separation of exosomes from other biological products. Each approach has advantages and limitations, and combining them for optimum exosome enrichment may be recommended (Thery et al., 2018). It is necessary to characterize exosomes thoroughly according to ISEV’s report for the validation of the isolation technique. Generally, Western Blot or ELISA are used for this purpose. The ISEV recommends identifying at least three positive and one negative protein markers. At least one transmembrane/lipid-bound protein (e.g., CD63, CD9, CD81) and one cytosolic protein (e.g., TSG101, ALIX) must be present as a positive protein marker. Single vesicle characterization requires imaging techniques (atomic force microscopy (AFM) and electron microscopy (EM)) and biophysical characterization (nanoparticle tracking analysis (NTA), tunable resistance pulse sensing (TRPS), dynamic light scattering (DLS), and flow cytometry (FC)) (Thery et al., 2018).

Bioengineered exosomes have greater therapeutic potential as delivery vehicles due their ability to transfer desired payloads and give better targeting specificity. To date, two key strategies for maximizing therapeutic efficacy of exosomes have been employed; (1) cargo engineering and, (2) surface engineering.

### Cargo engineering

Different medicinal substances, such as drugs, proteins, and nucleic acids, can be encapsulated by exosomes. Pre-loading (before separation) and post-loading (after isolation) are the two main types of cargo loading techniques. In pre-loading, therapeutic molecules can be endogenously packed into exosomes during the biogenesis stage by modifying parental cells. This can be accomplished by manipulating the genetics of parental cells. Parental cells can overexpress therapeutic miRNAs, siRNAs, mRNAs, proteins, and peptides by transfection, which then be encapsulated into exosomes. Another method is to directly incubate drugs with parental cells, resulting in drug-containing exosomes (Herrmann et al., 2021). The post-loading occurs after exosomes are isolated. Exogenous payloads are passively or actively loaded into exosomes. After direct co-incubation, hydrophobic drugs can be mixed with the exosome lipid bilayer membrane and incorporated into the surface. The hydrophobic nature of the payloads and the concentration gradient of the molecules determine this passive loading method, which usually results in a poor loading capacity (Liang Y. et al., 2021). Different active loading strategies for hydrophilic molecules have been developed to temporarily permeabilize the hydrophobic lipid barrier, either physically or chemically, allowing the passage of the drug into exosomes. Electroporation, sonication, freeze-thaw cycles, and extrusion are examples of physical techniques that entail brief disruption of the exosome membrane by external forces (Walker et al., 2019). Electroporation is currently the most popular method, particularly for RNA encapsulation. Chemical techniques, on the other hand, use transfection reagents or permeabilizers like saponin to help payloads enter the exosomes without disrupting its lipid bilayer structure (Haney et al., 2019).

### Surface engineering

**FIGURE 2:**
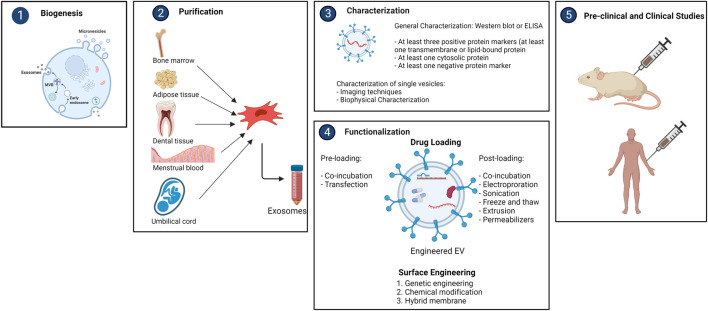
Overview of purification, characterization and functionalization of mesencyhmal stem cell-derived exosomes (created with BioRender).

Exosomes isolated from distinct cell origins have different surface molecules, indicating that they are selective for specific recipient cells. The biodistribution and tropism of exosomes can be influenced by changing their surface, particularly their protein composition. The major purpose of surface engineering is to give exosomes more targeting specificity, raising the local concentration of exosomes at desirable localizations while lowering unwanted systemic toxicity. Genetic engineering, chemical modification, and hybrid membrane engineering are the three types of surface engineering technologies (Liang W et al., 2021) (Figure 2).

## Mesenchymal stem cell-derived exosomes in cancer

Exosomes can be isolated from cell cultures or body fluids. The most common cell sources are MSCs, immune cells, and cancer cells. MSCs are the most abundant producer when compared to other cell sources, and they have a large expansion capacity for economically feasible exosome production (Kim et al., 2021). Additionally, MSCs can also be isolated from a variety of human tissues without having an ethical concern (Zhou et al., 2021). Numerous *in vivo* and *in vitro* studies demonstrate the immunoregulatory, pro-angiogenic, and tissue-regeneration properties of MSC-derived exosomes. For instance, MSC-derived exosomes alleviate the severity of myocardial injury (Ma J. et al., 2017); promote tissue damage repair (Zhang B. et al., 2015); and regulate the immune system (Ti et al., 2015). Other benefits include the prevention of acute tubular injury (Bruno et al., 2009), nerve injury (Drommelschmidt et al., 2017), and lung injury (Lee et al., 2012). Preclinical data have proven the safety of exosome therapy and scalability of their isolation methods from MSCs for clinical application. However, due to the lack of established cell culture conditions, suitable protocols for production, isolation, and storage of exosomes, optimal therapeutic dose and administration schedule, and reliable potency assays to assess the efficacy of exosome therapy, the use of MSC-derived exosomes in clinical settings is limited (Börger et al., 2017).

Recent studies have shown that MSC-derived exosomes play an important role in angiogenesis, tumor development, and tumor invasion. It is still unclear whether natural MSC-derived exosomes have beneficial or detrimental effects on tumors. Several studies have reported that natural MSC-derived exosomes enhanced tumor development. However, some studies suggested that these exosomes can prevent tumor progression. According to a prior study, the dual effect may be influenced by the origin of the MSC-derived exosomes, the dose and timing of the MSC injection, the kind of malignancy, and other parameters (Shojaei et al., 2019). Zhu et al. (2012) showed that exosomes released by MSCs could stimulate tumor growth *in vivo*. In xenograft mouse models of stomach and colon malignancies, exosomes generated from human bone marrow mesenchymal stem cells (hBMSCs) promoted tumor growth. However, exosomes had no similar effects on tumor cells *in vitro*. Angiogenesis-related molecular signaling pathway activation was detected *in vivo* and *in vitro* with increased VEGF and CXCR4 mRNA levels, which corresponded to enhanced vascular density in tumor tissues *in vivo*. Finally, they showed that stimulation of the ERK1/2 and p38 MAPK pathways by hBMSC-derived exosomes increased VEGF and CXCR4 expression in tumor cells, resulting in increased angiogenesis and hence tumor growth *in vivo* (Zhu et al., 2012). In non-small cell lung cancer (NSCLC), MSC and MSC-derived exosomes promote malignancy by triggering epithelial mesenchymal transition, migration, autophagy, and also inhibiting apoptosis through the activation of the AMPK signaling pathway (Wang et al., 2022). In hepatocellular carcinoma (HCC) cells, MSC-derived exosomes increase proliferation, invasion, sphere formation ability and suppress apoptosis through TMBIM6. As a result of silencing TMBIM6, viability, sphere formation, invasion, epithelial mesenchymal transition and PI3K/AKT signaling pathway are suppressed, and apoptosis is triggered (Shang et al., 2022). Adipocyte-derived exosomes differentiated from MSC in breast cancer promote cell proliferation and migration, and also inhibit apoptosis via the Hippo signaling pathway. Suppression of the signaling pathway blocks the growth-promoting effect of adipocyte exosomes (Wang S. et al., 2019).

On the contrary, Wu et al. (2013) found that human umbilical cord Wharton’s jelly mesenchymal stem cells (hWJMSCs)-derived exosomes could induce apoptosis and cell cycle arrest in T24, a bladder cancer cell line, by increasing the expression of caspase-3 and decreasing the phosphorylation of Akt. According to a study by Kalimuthu et al. (2016) treatment with MSC-derived extracellular vesicles led lung cancer cells to undergo apoptosis.

## Functions of miRNA loaded mesenchymal stem cell-derived exosomes in cancer

miRNAs are a family of short single-stranded non-coding RNAs that regulate gene expression in target cells. They range in length from 20 to 25 nucleotides (Leavitt et al., 2019). miRNAs act at the 3′UTR of mRNAs to downregulate their translation or cause their degradation as part of the RNA-induced silencing complex (RISC) (Gu et al., 2009). miRNA expression can be altered due to many reasons such as germline and somatic mutations in miRNA genes, amplification or deletion of miRNA genes, epigenetic regulation in miRNA locus, changes in miRNA biogenesis mechanisms, editing and chemical modifications of miRNAs. These dysregulations result in up- or downregulation of miRNAs and predispose to the formation of many diseases, including cancer (Urbanek-Trzeciak et al., 2020). Under specific circumstances, miRNAs can act as tumor suppressors or oncogenes. It has been demonstrated that dysregulated miRNAs have an impact on the characteristics of cancer, including maintaining proliferative signaling, avoiding growth inhibitors, resisting cell death, triggering invasion and metastasis, and promoting angiogenesis (Table 1). miRNAs have been identified as possible biomarkers for the diagnosis and prognosis of human cancers and therapeutic targets (Peng and Croce, 2016).

**TABLE 1:** Examples of miRNAs and their roles in different cancers.

Cancer type	miRNA	Expression	Target	Pathway	Effect	References
Brain Cancer	miR-7	Downregulated	EGFR, PI3K- Akt EGFR, IRS1, IRS2	EGFR, PTEN-PI3K-Akt IGF-1R/Akt	Cell growth, cell cycle arrest Invasion, proliferation, cell cycle, survival/cell death	Liu Z. et al. (2014), Matos et al. (2018)
	miR-101	Downregulated	SOX9	Akt, Wnt, BMI1	Proliferation, migration, invasion	Liu et al. (2017)
	miR-29a/b/c	Downregulated	CDC42	CDC42-PAK	Migration, invasion	Shi et al. (2017)
	miR-146b-5p	Downregulated	TRAF6	TRAF6-TAK1	Cell proliferation, apoptosis resistance	Liu et al. (2015)
	miR-181c	Downregulated	NOTCH2	NOTCH	Tumor progression	Ayala-Ortega et al. (2016)
	miR-320a	Downregulated	SND1, β-catenin	TGFβ1	Cell proliferation, invasion, migration	Li et al. (2017)
	miR-21	Upregulated	EGFR, Akt, cyclin D, Bcl-2	EGFR, Akt	Apoptosis, TMZ resistance	Zhou et al. (2010), Wong et al. (2012)
	miR-221 miR-222	Upregulated	SOCS3	JAK/STAT	Invasion, migration, proliferation, angiogenesis	Xu C. H. et al. (2019)
	miR-10b	Upregulated	PTEN, p53, BIM E-cadherin, Apaf-1, PTEN/TGF-β1	TGF-β	Growth, invasion, apoptosis Proliferation, migration, EMT.	Sun et al. (2019), Ma C. et al. (2017)
	miR-181b	Upregulated	KPNA4	EMT	Growth, invasion, proliferation	Wang et al. (2015)
	miR-141	Upregulated	Jagged1	NOTCH	Growth	Gao et al. (2017)
Head and Neck Cancer	let-7c	Downregulated	IL-8		Radio-/chemoresistance	Peng C. Y. et al. (2018)
	miR-101	Downregulated	EZH2 CDK8	Wnt/β-catenin	Metastasis, EMT Tumorigenesis	Chen L. et al. (2019), Li et al. (2015)
	miR-124	Downregulated	STAT3	JAK/STAT	Tumor growth and metastasis	Xu et al. (2016)
	miR-let-7e	Downregulated	HMGB1	NF-κB	Migration, invasion	Ding C. et al. (2019)
	miR-206	Downregulated	MAP4K3	p38, JNK	Cell proliferation, apoptosis, multidrug resistance	Liu et al. (2019)
	miR-30a miR-379	Downregulated	DNMT3B	Retinoic acid pathway	Growth	Shiah et al. (2020)
	miR-125a	Upregulated	p53		Cell proliferation, migration, invasion	Chen J. et al. (2019)
	miR-134	Upregulated	PDCD7 WWOX		E-cadherin expression Suppressor inhibition	Peng S. Y. et al. (2018), Liu C. J. et al. (2014)
	miR-196b	Upregulated	PCDH-17		Cell proliferation, migration, and invasion	Luo M. et al. (2019)
	miR-144	Upregulated	mTOR	PI3K/Akt/mTOR	Cell proliferation, clonogenicity, migration, invasion, tumor formation	Shabani et al. (2018)
Breast Cancer	miR-126	Downregulated	VEGFA, PIK3R2	VEGF/PI3K/Akt	Angiogenesis	Zhu et al. (2011)
	miR-204	Downregulated	PI3K-α, c-SRC, VEGF, FAK, RAF1, MAPK	PI3K/AKT, RAF1/MAPK, VEGF, FAK/SRC	Angiogenesis	Salinas-Vera et al. (2018)
	miR-720	Downregulated	ADAM8	ERK	Metastasis	Das et al. (2016)
	miR-205	Downregulated	ZEB1, ZEB2, HER3, AMOT, erbB2/erbB3		Proliferation, invasion, metastasis	Wang et al. (2013), Zhang and Fan, (2015), Huo et al. (2016)
	miR-200 family	Downregulated	ZEB2, E-cadherin		Metastasis, invasion	Liu et al. (2018), Rogers et al. (2019)
	miR-203a-3p	Downregulated	ZEB2		Metastasis, invasion	Fahim et al. (2020)
	miR-1-3p	Downregulated	K-RAS, MALAT1		Proliferation, apoptosis	Chou et al. (2016), Jin et al. (2016), Fahim et al. (2020)
	miR-210	Upregulated	HRAS, PTK2, SHC1, HIF1a	Hypoxia VEGF signaling	Development of cancer, angiogenesis	Foekens et al. (2008)
	miR-182	Upregulated	FBXW7	HIF-1α- VEGF-A	Proliferation, angiogenesis	Chiang et al. (2016)
	miR-155	Upregulated	VHL	VHL/HIF-1α/VEGF	Angiogenesis	Kong et al. (2014)
	miR526b miR655	Upregulated	VEGFA, VEGFC, VEGFD, CD31, LYVE1	PI3K/Akt	Angiogenesis	Hunter et al. (2019)
	miR-20b	Upregulated	PTEN	PTEN-PI3K-Akt	Progression, angiogenesis	Zhou et al. (2014)
	miR-155 miR-203 miR-125a	Upregulated	SOCS1, SOCS3, STAT3, PIAS3, IL-6, IL-6R	JAK/STAT3		Lei et al. (2016), Ru et al. (2011), Park and Kim, (2019)
Gastrointestinal Cancer	miR-28-5p	Downregulated	AKT		Proliferation, migration	Xiao et al. (2018)
	miR-7	Downregulated	RelA/p65 Raf-1	NF-κB	Metastasis, tumor development, angiogenesis	Ye et al. (2019), Lin J. et al. (2020)
	miR-1299	Downregulated	ARF6		Proliferation, apoptosis, migration, invasion	Qiu et al. (2022)
	miR-223-3p	Downregulated				Zhou et al. (2017)
	miR-339-5p	Downregulated	Cdc25A			Luo A. et al. (2019)
	miR-148a-3p miR-181a-5p	Downregulated				Lin Z. et al. (2020)
	miR-497	Downregulated			Differentiation, lymph node metastasis	Zou G. et al. (2019)
	miR-100	Downregulated				Stroese et al. (2018)
	miR-181a	Upregulated	Caprin-1		Proliferation, apoptosis, invasion, metastasis	Lu et al. (2019)
	miR-653-5p	Upregulated	SOCS6-STAT3	JAK2/STAT3 pathway	Proliferation, metastasis	Li Z. et al. (2021)
	miR-1301-3p	Upregulated	SIRT1		Proliferation, cell cycle, tumorigenesis	Luo et al. (2021)
	miR-106a miR-18a miR-20b miR-486-5p miR-584	Upregulated				Zhou et al. (2017)
	miR-34a-5p	Upregulated				Lin Z. et al. (2020)
	miR-199a-3p	Upregulated			Depth of invasion	Nonaka et al. (2014)
	miR-103 miR-720	Upregulated			Differentiation, lymph node metastasis	Nonaka et al. (2015)
	miR-19a-3p miR-19b-3p miR-25-3p miR-192-5p miR-223-3p	Upregulated				Zou X. et al. (2019)
Genitourinary Cancer	miRNA-199a-3p	Downregulated	Cyclin D1, c-Myc, mTOR EGFR		Proliferation, clonal expansion, regeneration	Liu et al. (2016)
	miRNA-203	Downregulated	IRS-1	ERK	Cell proliferation, cell cycle	Meng et al. (2020)
	miRNA-218	Downregulated	GAB2	PI3K/Akt/GSK-3β	Cell proliferation, migration	Tian et al. (2020)
	miRNA-1	Downregulated	c-Met	Akt/mTOR	Cell survival, proliferation	Gao et al. (2019)
	miRNA-31-5p	Downregulated	14–3-3 ε	PI3K/AKT/Bcl-2	Cell survival, proliferation	Zhao et al. (2020)
	miRNA-381	Downregulated	RELN	PI3K/Akt/mTOR	Autophagy, apoptosis	Liao and Zhang, (2020)
	miRNA-125b	Upregulated	p14ARF	p53	Cell proliferation	Amir et al. (2013)
	miRNA-486-5p	Upregulated		SMAD2/TGF- β PTEN/PI3K FoxO	Proliferation, development, pathogenesis	Yang et al. (2017)
	miRNA-4534	Upregulated		PTEN/PI3K/Akt	Migration, apoptosis	Nip et al. (2016)
Gynecologic Cancer	let-7d-5p	Downregulated	HMGA1	p53	Proliferation, chemosensitivity	Chen Y. N. et al. (2019)
	miR-101-5p	Downregulated	CXCL6		Colony formation, invasion, migration	Shen et al. (2019)
	miR-132	Downregulated	SMAD2		Lymph node metastasis	Zhao J. L. et al. (2015)
	miR-138-5p	Downregulated	SIRT1		Tumorigenesis, metastasis	Ou et al. (2018)
	miR-148b	Downregulated	CASP3		Cell proliferation, invasion, apoptosis	Mou et al. (2016)
	miR-508 miR-509–2 miR-526b	Downregulated	p53,SMAD4, NF-κB-1, MMP1, NOTCH1, SMAD4		Migration, invasion, lymph node metastasis, tumor progression	Chen et al. (2018)
	miR-16–1	Upregulated	CycE1		Controls the transition of cells from G1 to S phase	Zubillaga-Guerrero et al. (2015)
	miR-20a	Upregulated	TIMP2, ATG7		Lymph node metastasis, invasion	Zhao S. et al. (2015)
	miR-20b	Upregulated	TIMP2		Regulates the cytoskeleton and activates EMT, migration, invasion	Cheng et al. (2017)
	miR-27b	Upregulated	CDH11		Proliferation, cell cycle transition from G1 to S phase, migration, invasion	Yao et al. (2016)
	miR-106b-5p	Upregulated	GSK3B, VEGFA, PTK2	PI3K-Akt	Lymph node metastasis	Yi et al. (2018)
Hematologic Cancer	miR-3173	Downregulated	PTK2		Proliferation, migration, invasion	Tian et al. (2017)
	miR-181a	Downregulated	Smad7	TGF-β1	Proliferation, apoptosis, diagnostic sensitivity	Nabhan et al. (2017)
	miR-142-3p	Downregulated	MLL-AF4, HOXA7, HOXA9, HOXA10		Cell proliferation	Dou et al. (2013)
	hsa-miR-103a-3p hsa-miR486-3p	Downregulated	HOXA7, S100A10		Cell growth, motility, cell cycle progression, differentiation, Poor outcomes, chemoresistance	Huang et al. (2020)
	miR-21	Upregulated	PDCD4, PTEN, TPM1		Cell growth, invasion, angiogenesis, metastasis	Labib et al. (2017)
	miR-339-5p	Upregulated	BCL2L11, Bax, FGFR1		Cell cycle progression, apoptosis	Hu et al. (2018)
	miR-125b miR-17 miR-181b	Upregulated	PPP1CA, BTG2, PTEN		Proliferation, apoptosis	Vafadar et al. (2019)
	miR-187-5p	Upregulated	DKK2	Wnt/β-catenin	Proliferation, apoptosis	Lou et al. (2016)

Two types of miRNA-based approaches can be used to change the expression levels of target genes for therapeutic purposes: (a) miRNA suppression therapy when the target gene is downregulated and (b) miRNA replacement therapy when the target gene is upregulated. Usually, the reticuloendothelial system and the ribonucleases present in the blood rapidly degrade naked RNA. The stability of oligonucleotides can be improved by chemical modifications for *in vivo* delivery. Antisense oligonucleotide (ASO) technology was developed for studying miRNA, and the ASOs that are used to silence miRNA are called anti-miRNA oligonucleotides (AMOs) (Zhang and Farwell, 2008).

miRNA suppression therapy can remove miRNA suppression on the target mRNA, thus increasing the mRNA expression level. AMOs bind to the miRNA sense strand, block interactions between miRISC and its target mRNA, prevent the degradation of the mRNA, and thus allow the mRNA to be translated. In miRNA replacement therapy, miRNA mimics, synthetic double-stranded miRNA-like RNA molecules, can stimulate endogenous miRNAs and bind to mRNA of the target gene, resulting in posttranscriptional suppression (Fu et al., 2019). Since cancer is related with the deregulation of multiple genes and miRNAs, it is commonly accepted that focusing on just one target is insufficient for an effective treatment. Therefore high target specificity has been replaced with multi-specificity. In that regard, miRNA-based therapies are an advantage since they affect the regulatory sequence, commonly functioning on an entire pathway or even several pathways rather than just one gene (Baumann and Winkler, 2014).

Because of their negative charge and hydrophilic nature, miRNAs are difficult to cross the cell membrane. Additionally, they are destroyed after entering the body. Therefore, exosomes can serve as excellent carriers for miRNAs (Zhang et al., 2022). There are two methods for miRNA enrichment/loading in exosomes. The first strategy involves creating a cell line that overexpresses the desired therapeutic miRNA. The cell line then displays a high level of miRNA in their cytoplasm, followed by exosome secretion containing therapeutic miRNA. The second strategy involves separating exosomes from the source (cell lines or body fluids) and then loading them with selected miRNA by using chemical or physical approaches. Since it is widely known that increasing the quantity of miRNA in the cytosol may increase their passive loading in exosomes, it is possible to transfect a designed miRNA into cells for exosomal therapy. Choosing the right cell type is one of the requirements for transfection. Although MSCs are the most commonly used “biofactories” for producing exosomes with loaded miRNA, there are some limitations in their utilization for therapeutic purposes. Initially, the cell system should be selected carefully according to the purpose of miRNA-loading. The disease being studied, the dynamics of communication between exosome-producing cells and the recipient cell, the rate of exosome secretion, and the capacity of exosomes to uptake exogenous therapeutic miRNAs should also be considered (Munir et al., 2020). Exosomes essentially have proteins on their surface, such as tetraspanins (CD-81, -82, -37, and CD-63), membrane trafficking proteins, cytoskeletal proteins, and two members of the Endosomal Sorting Complex Required for Transport (ESCRT) pathway, namely Alix and Tumor Susceptibility Gene 101 (TSG-101). The propensity of these proteins to target particular tissues is modest. Additionally, these proteins enable exosomes to accumulate in the liver, kidney, and spleen. They can be also eliminated through bile, renal filtration, and reticuloendothelial phagocytosis (Xitong and Xiaorong, 2016). Therefore, it is strongly advised to change the surface of exosomes in order to improve precise targeting and decrease the clearance rate. This can be accomplished by directly or genetically altering the exosome membrane proteins. Exosome surfaces can be directly altered using non-covalent or covalent techniques. In the non-covalent technique, exosomes and protein are combined. The covalent technique, on the other hand, involves the attachment of a peptide with covalent bonding. However, it remains to be unclear how effective these methods are for developing miRNA-enriched exosomes for targeted therapy. Both techniques have the potential for chemical contamination and have varying degrees of modification efficacy. Additionally, non-covalent attachment may dissociate under physiologic conditions (Hu et al., 2020). Genetic alteration involves producing a particular protein on the exosome surface which results in more homogenous population and sustained target specificity. It is more expensive than a direct approach. Additionally, it raises safety issues, which makes it unsuitable for clinical uses (Ohno et al., 2013).

The effect of miRNAs carried by MSC-derived exosomes in tumor treatment is contradictory, with some research claiming that they can stimulate tumor growth and others claiming that they can repress tumor growth. In osteosarcoma, miR-208a in MSC-derived exosomes increased tumor growth by downregulating programmed cell death and activating the ERK1/2 pathway (Qin et al., 2020). Furthermore, MSC-exosome-derived miR-142-3p and miR-146a have been shown to stimulate tumor growth *via* many mechanisms (De Veirman et al., 2016; Li and Li, 2018). Similarly, miR-146a can enhance the progression of multiple myeloma, validating this concept (De Veirman et al., 2016).

On the other hand, anti-tumor effects of miRNA carrying MSC-derived exosomes have been shown by different groups (Kang et al., 2015; Renjie and Haiqian, 2015; Gopalan et al., 2018). In prostate cancer, human bone marrow MSC-derived exosomal miR-143 has been shown to inhibit cell proliferation, invasion, metastasis, and tumor growth (Che et al., 2019). miR-23b in MSC-derived exosomes can prevent tumor development, keep tumors dormant, improve patient’s life quality, and lengthen survival time (Ono et al., 2014). In hepatocellular carcinoma, MSC-derived exosomes transfected with miR-122 can improve drug sensitivity (Lou et al., 2015). miR-34c in MSC has been proven to improve tumor sensitivity to radiotherapy in addition to enhancing chemical sensitivity (Wan et al., 2020). This shows that MSC-exosomes can be used in combination with conventional cancer treatments such as chemotherapy and radiotherapy.

miRNAs in MSC-derived exosomes have received a great deal of interest recently, and they are being studied largely for tumor inhibition. These studies differ from each other in terms of the cancer type of interest, selected MSC subtype, the way of miRNAs is transferred to MSCs, preferred miRNA and target genes according to the cancer type. The general approach in studies is to first detect and confirm miRNA and target genes that negatively regulate each other in bioinformatic studies or healthy/patient samples, and then detect alteration in the proliferation, apoptosis, migration and invasion capacities of cancer cells after administration *in vitro* and *in vivo*. The general conclusion reached is that miRNA transfer with MSC-derived exosomes has positive effects. However, it is emphasized that such studies are at a preclinical stage, the data on the mechanism of action are still insufficient, and therefore studies should continue in order to reveal the mechanisms. We summarized the studies on the use of MSC-derived exosomes as vehicles for the delivery and restoration of miRNAs in Table 2, with the goal of developing an effective therapeutic strategy for various malignancies.

**TABLE 2:** Effects of miRNAs delivered by mesencymal stem cell-derived exosomes in different cancer types.

Type of cancer	Source of MSC	miRNA	Target gene/Pathway	Effects	References
Brain Cancer					
Glioma		miR-584	CYP2J2, MMP-2, Bcl-2, Bax exp.	↓ proliferation, invasion, metastasis, ↑ apoptosis	Kim et al. (2018)
	Bone marrow (mice)	miR-133b	EZH2 exp. Wnt/β-catenin signaling pathway	↓ proliferation, invasion, migration	Xu H. et al. (2019)
	Bone marrow (human)	miR-34a	SIRT1 exp.	↑ cellular senescence	Li Q. et al. (2019)
	Bone marrow (human)	miR-199a	AGAP2 exp.	↓ proliferation, invasion, migration, ↓tumor growth (*in vivo*), ↑ chemosensitivity to temozolomide (*in vivo*)	Yu et al. (2019)
Glioblastoma multiforme	Wharton’s jelly (human)	miR-124	CDK6 exp.	↓ migration, ↑ chemosensitivity to temozolomide	Sharif et al. (2018)
	Adipose tissue (human)	miR-4731		↓ proliferation stimulation of cell cycle arrest, apoptosis	Allahverdi et al. (2020)
	Bone marrow (human)	miR-512-5p	JAG1 exp. Notch signaling pathway	↓ proliferation stimulation of cell cycle arrest, ↓ tumor growth (*in vivo*) prolongation of survival (*in vivo*)	Yan et al. (2021)
	Bone marrow (human)	miR-30c	IL-6 exp	↓ migration, invasion, ↑ apoptosis	Mahjoor et al. (2021)
Neuroblastoma	Adipose tissue (human)	miR-124		↓ proliferation, ↑ apoptosis stimulate neuronal differentiation	Sharif et al. (2021)
Head and Neck Cancer					
Oral cancer	Bone marrow (human)	miR-101-3p	COL10A1 exp.	↓ proliferation, invasion, migration, ↓tumor growth (*in vivo*)	Xie et al. (2019)
Oral leukoplakia	Bone marrow (mice)	miR-185	Akt, caspase-3 and 9 exp.	↓severity of inflammation (*in vivo*), ↓number of dysplasia in the OPMD tissue (*in vivo*), ↑ apoptosis	Wang L. et al. (2019)
Thyroid cancer	Umbilical cord (human)	miR-30c-5p	PELI1, Ki-67, MMP-2 exp., PI3K-AKT signaling pathway	↓ proliferation, migration, ↓tumor growth (*in vivo*)	Zheng et al. (2022)
Breast Cancer					
	Bone marrow (mice)	LNA-antimiR-142-3p	miR-150, APC, P2X7R exp. Wnt signaling pathway	Penetration to the tumor site (*in vivo*), ↓ reduction of clone-formation, tumor-initiating abilitiy	Naseri et al. (2018), Naseri et al. (2020)
	Umbilical cord (human)	miR-148b-3p	TRIM59 exp.	suppressive effect on the progression, antitumor effect (*in vivo*)	Yuan et al. (2019)
	Adipose tissue (human)	miR-145	ROCK1, MMP9, ERBB2, TP53 exp.	↓ metastasis, ↑ apoptosis	Sheykhhasan et al. (2021)
	Umbilical cord (human)	miR-3182	mTOR, S6KB1 exp.	↓ proliferation, migration, ↑ apoptosis	Khazaei-Poul et al. (2021)
	Adipose tissue	miR-381	Wnt signaling pathway	↓ proliferation, migration, invasion, ↓ epithelial mesenchymal transition, ↑ apoptosis	Shojaei et al. (2021)
Gastrointestinal Cancer					
Esophageal squamous cell carcinoma	Umbilical cord (human)	miR-375	ENAH	↓ proliferation, migration, invasion, tumorsphere formation, ↑ apoptosis, ↓tumor growth (*in vivo*)	He et al. (2020)
Gastric cancer	Umbilical cord (human)	miR-6785-5p	INHBA exp	↓ angiogenesis, metastasis	Chen et al. (2021)
Pancreatic ductal adenocarcinoma	Umbilical cord (human)	miR-145-5p	Smad 3 exp	↓ proliferation, invasion, ↑ apoptosis, cell cycle arrest, ↓ tumor growth (*in vivo*)	Ding Y. et al. (2019)
Liver cancer	Adipose tissue (human)	miR-122	Genes involved in drug resistance or sensitivity	↑ susceptibility to chemotherapeutic drugs, ↑ anticancer activity of sorafenib (*in vivo*)	Lou et al. (2015)
	Adipose tissue (human)	miR-199a	mTOR signaling pathway	↑ sensitivity to doxorubicin	Lou et al. (2020)
Genitourinary Cancer					
Prostate cancer	Bone marrow (human)	miR-205	RHPN2 exp	↓ proliferation, invasion, metastasis, ↑ apoptosis	Jiang et al. (2019)
Bladder cancer	Umbilical cord (human)	miR-139-5p	PRC1	↓ development of bladder cancer	Jia et al. (2021)
Gynecologic Cancer					
Endometrial cancer	Umbilical cord (human)	miR-302a	cyclin D1 exp. AKT signaling pathway	↓ proliferation, migration	Li X. et al. (2019)
		miR-499a-5p	VAV3 exp	↓ proliferation, endothelial cell tube formation, ↓ tumor growth and angiogenesis (*in vivo*)	Jing et al. (2020)
Cervical cancer	Bone marrow (human)	miR-144-3p	CEP55 exp	↓ proliferation, migration, invasion, ↑ apoptosis	Meng et al. (2021)
Ovarian cancer	Bone marrow (mice)	miR-424	MYB, VEGF, VEGFR exp.	↓ proliferation, migration, invasion of ovarian cancer cells, ↓ proliferation, migration, invasion, tube formation of human umbilical vein endothelial cells, ↓ tumorigenesis, angiogenesis (*in vivo*)	Li P. et al. (2021)
Hematologic Cancer					
Acute myeloid leukemia	Bone marrow (human)	miR-222-3p	IRF2 exp. IRF2/INPP4B signaling pathway	↓ proliferation, ↑ apoptosis	Zhang et al. (2020)
	Bone marrow (human)	miR-26a-5p	GSK3 exp. Wnt/β-catenin signaling pathway	promoting effect on AML progression	Ji et al. (2021)
Other cancer types					
Bone cancer	Bone marrow (human)	miR-143,		↓ proliferation, migration,	Shimbo et al. (2014),
	Bone marrow (mice)	miR-9-5p	REST, cytokine, MOR exp.	alleviation of bone cancer pain by modulating neuroinflammation in the central nervous system	Zhu et al. (2020)
Lung cancer	Bone marrow	miR-328-3p	NF2 exp. Hippo signaling pathway	promote formation and progression of cancer	Liu et al. (2021)
	Umbilical cord (human)	miR-320a	SOX4 exp. SOX4/Wnt/β-catenin axis	Antitumor effect	Xie and Wang, (2021)

## Conclusion

While significant progress has been made in the fight against cancer, it remains a leading cause of mortality in the twenty-first century, necessitating a greater understanding of the biology of cancer cells and their environment in order to create novel therapeutic options. Over the last three decades, researchers and clinicians have mostly concentrated on identifying cancer-specific targets and developing targeted medicines that can effectively destroy cancer cells while sparing their normal counterparts, decreasing undesired side effects. A variety of intriguing and very effective small compounds targeting cancer-specific mutations and/or altered signal transduction pathways that control cancer cell proliferation and survival have been developed as a result of this global endeavor.

MSC-derived exosomes have been identified as significant mediators in the therapeutic benefits of MSCs. MSC-derived exosomes can promote or inhibit tumor growth, but engineered MSC-derived exosomes are implicated in the suppression of cancer formation and progression by the delivery of numerous therapeutic compounds, including miRNAs. Dysregulation of miRNAs is thought to be involved in the initiation and progression of tumors. Furthermore, promising results show that restoring these regulatory miRNAs can be used as a therapeutic method in cancer treatment. Replacement of these molecules can contribute to the inhibition of cell proliferation, invasion, migration, and metastasis, along with increased sensitivity to chemotherapeutic drugs and activation of apoptosis through direct control of their target genes.

To summarize, recent findings have confirmed the capacity of MSC-derived exosomes to transport therapeutic miRNAs in a variety of malignancies, indicating that this approach is novel and extremely promising in the treatment of cancer. Despite the fact that MSCs have been shown to have anticancer properties, there have also been some conflicting claims about their roles in tumor growth. Hence, their potential in tumor progression should also be considered.
